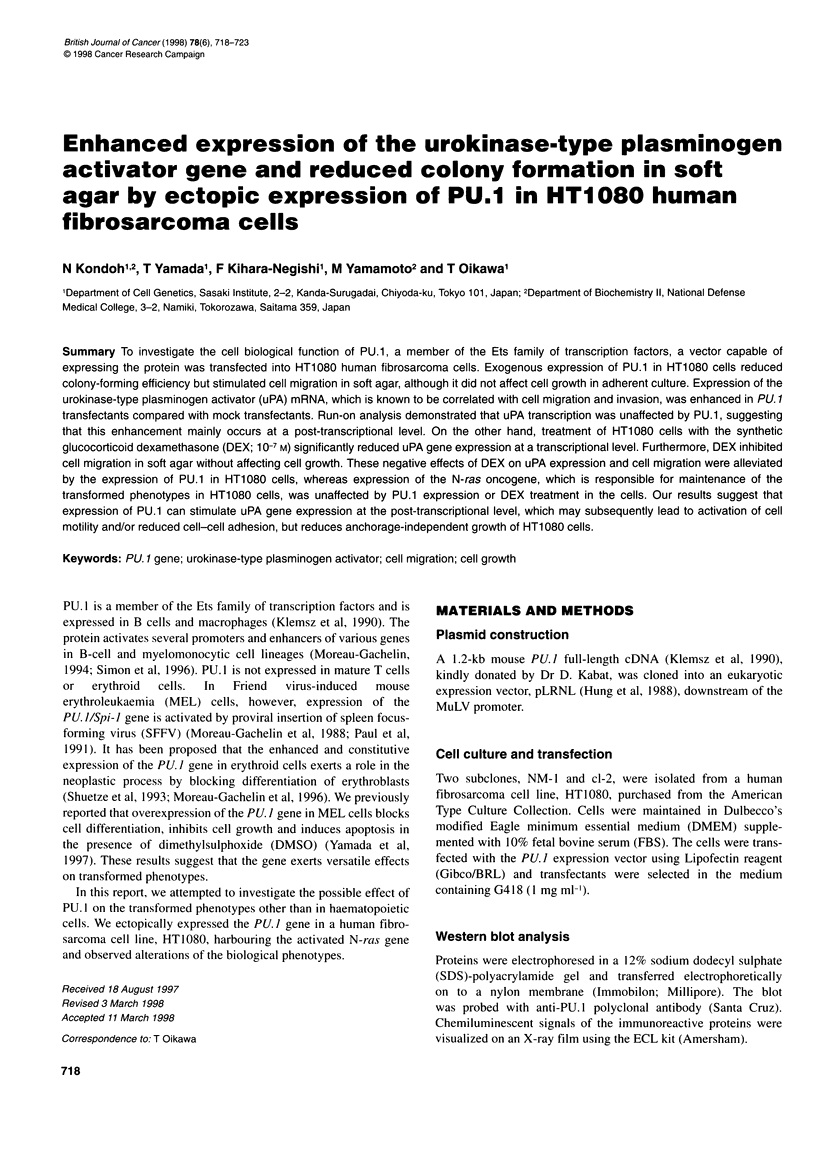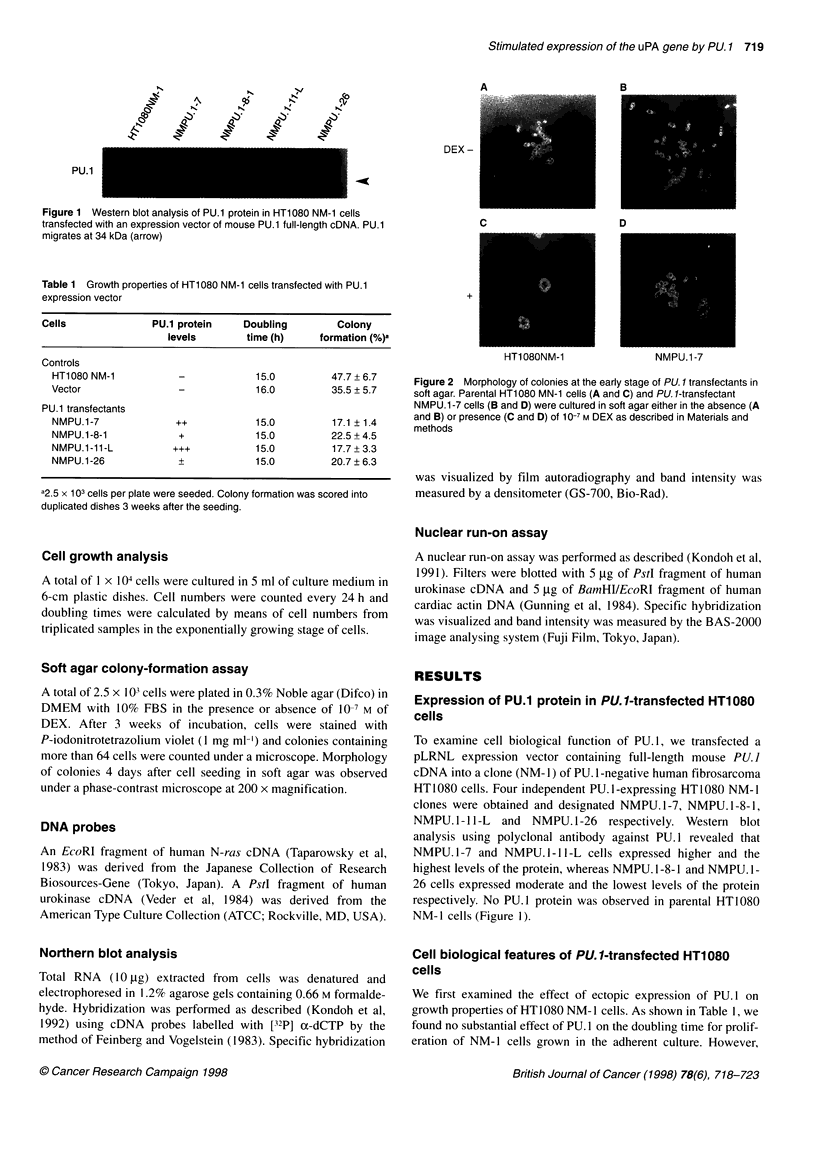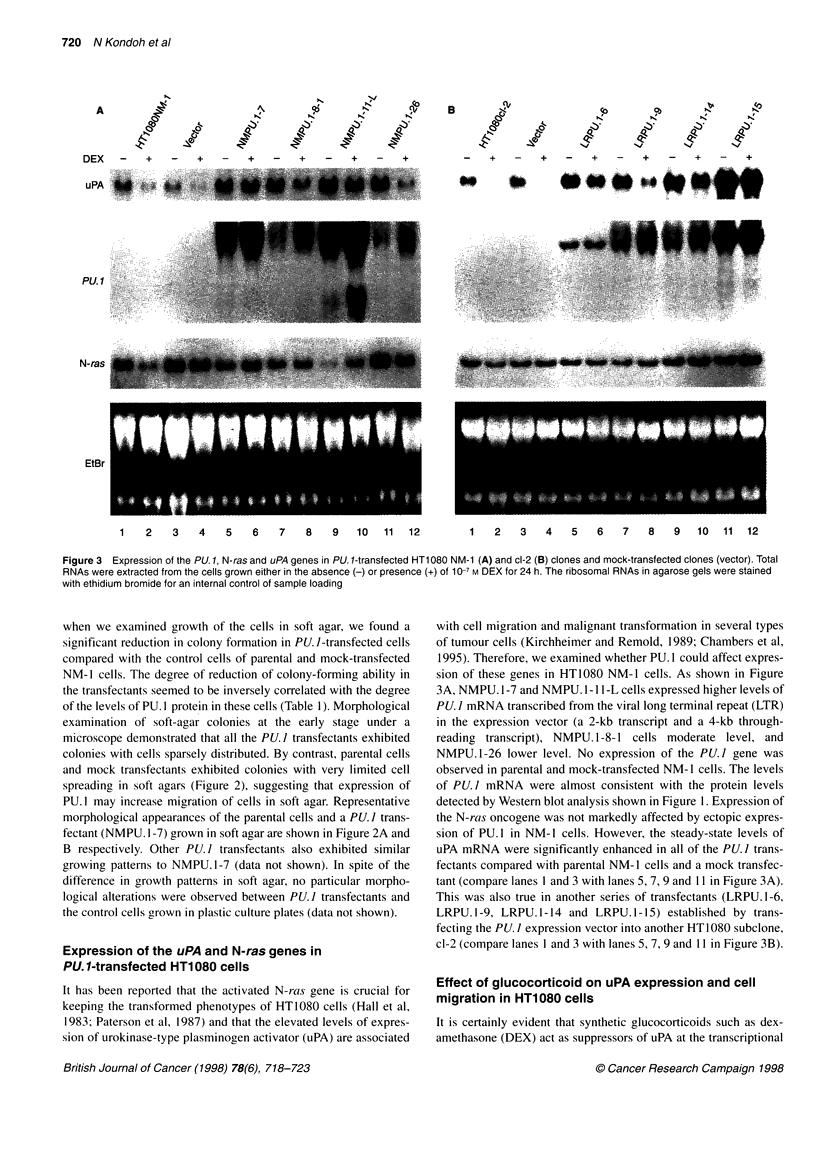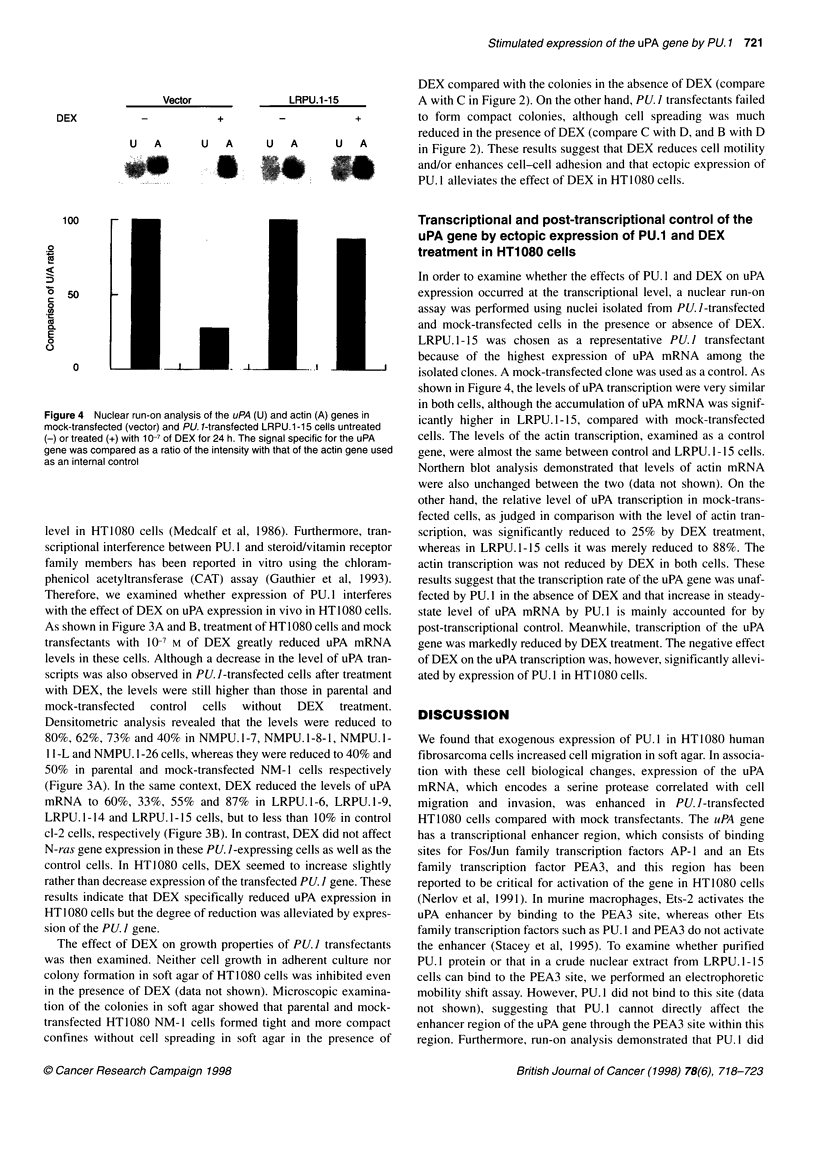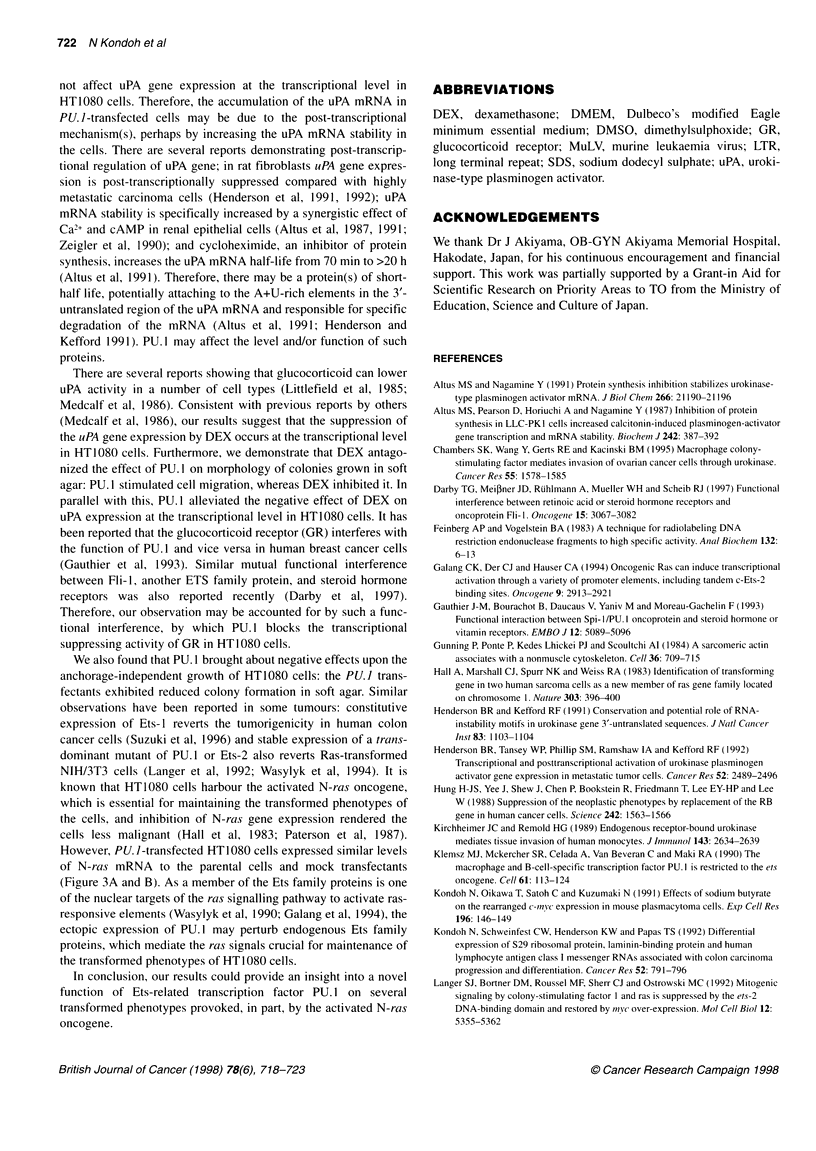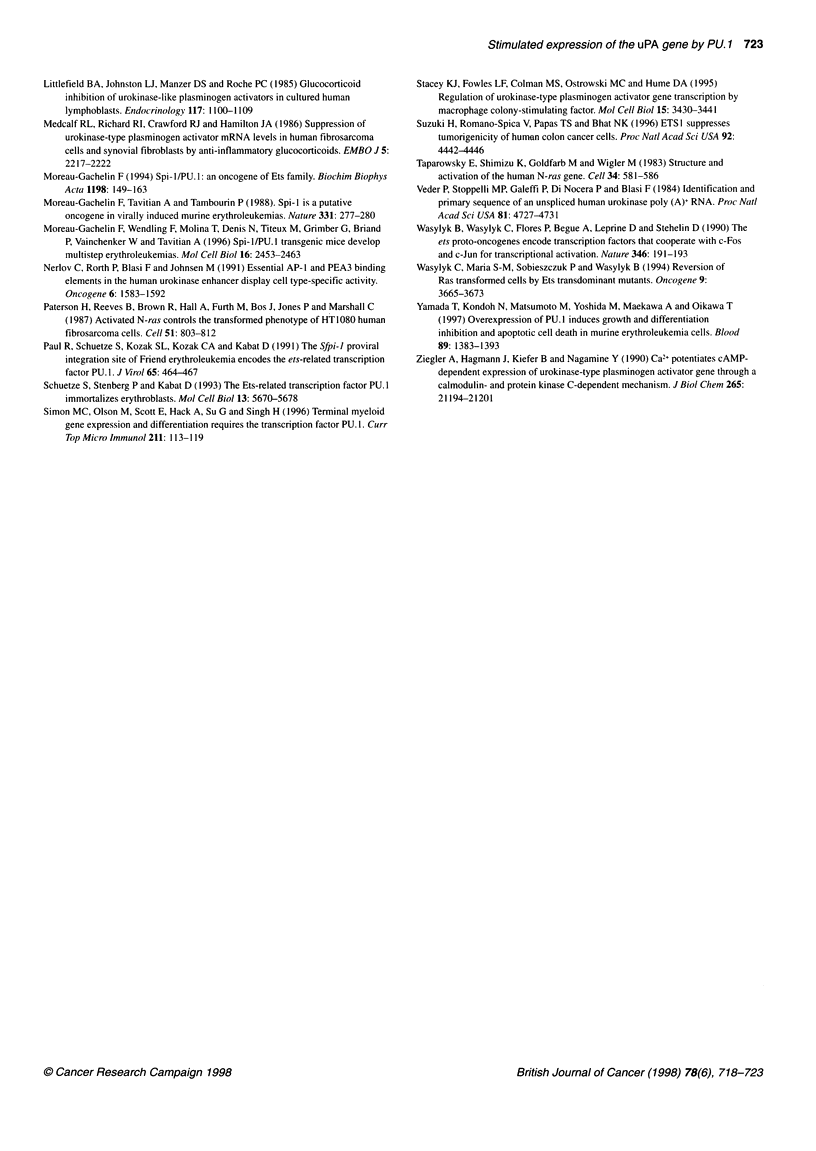# Enhanced expression of the urokinase-type plasminogen activator gene and reduced colony formation in soft agar by ectopic expression of PU.1 in HT1080 human fibrosarcoma cells.

**DOI:** 10.1038/bjc.1998.567

**Published:** 1998-09

**Authors:** N. Kondoh, T. Yamada, F. Kihara-Negishi, M. Yamamoto, T. Oikawa

**Affiliations:** Department of Cell Genetics, Sasaki Institute, Tokyo, Japan.

## Abstract

**Images:**


*British Journal of Cancer* (1998) **78**(6), 718–723
© 1998 Cancer Research Campaign

### Enhanced expression of the urokinase-type plasminogen activator gene and reduced colony formation in soft agar by ectopic expression of PU.1 in HT1080 human fibrosarcoma cells

**N Kondoh[1,2], T Yamada[1], F Kihara-Negishi[1], M Yamamoto[2] and T Oikawa[1]**

[1]Department of Cell Genetics, Sasaki Institute, 2-2, Kanda-Surugadai, Chiyoda-ku, Tokyo 101, Japan; [2]Department of Biochemistry II, National Defense Medical College, 3-2, Namiki, Tokorozawa, Saitama 359, Japan

**Summary** To investigate the cell biological function of PU.1, a member of the Ets family of transcription factors, a vector capable of expressing the protein was transfected into HT1080 human fibrosarcoma cells. Exogenous expression of PU.1 in HT1080 cells reduced colony-forming efficiency but stimulated cell migration in soft agar, although it did not affect cell growth in adherent culture. Expression of the urokinase-type plasminogen activator (uPA) mRNA, which is known to be correlated with cell migration and invasion, was enhanced in *PU.1* transfectants compared with mock transfectants. Run-on analysis demonstrated that uPA transcription was unaffected by PU.1, suggesting that this enhancement mainly occurs at a post-transcriptional level. On the other hand, treatment of HT1080 cells with the synthetic glucocorticoid dexamethasone (DEX; $10^{-7}$ M) significantly reduced uPA gene expression at a transcriptional level. Furthermore, DEX inhibited cell migration in soft agar without affecting cell growth. These negative effects of DEX on uPA expression and cell migration were alleviated by the expression of PU.1 in HT1080 cells, whereas expression of the N-*ras* oncogene, which is responsible for maintenance of the transformed phenotypes in HT1080 cells, was unaffected by PU.1 expression or DEX treatment in the cells. Our results suggest that expression of PU.1 can stimulate uPA gene expression at the post-transcriptional level, which may subsequently lead to activation of cell motility and/or reduced cell–cell adhesion, but reduces anchorage-independent growth of HT1080 cells.

**Keywords:** *PU.1* gene; urokinase-type plasminogen activator; cell migration; cell growth

PU.1 is a member of the Ets family of transcription factors and is expressed in B cells and macrophages (Klemsz et al, 1990). The protein activates several promoters and enhancers of various genes in B-cell and myelomonocytic cell lineages (Moreau-Gachelin, 1994; Simon et al, 1996). PU.1 is not expressed in mature T cells or erythroid cells. In Friend virus-induced mouse erythroleukaemia (MEL) cells, however, expression of the *PU.1/Spi-1* gene is activated by proviral insertion of spleen focus-forming virus (SFFV) (Moreau-Gachelin et al, 1988; Paul et al, 1991). It has been proposed that the enhanced and constitutive expression of the *PU.1* gene in erythroid cells exerts a role in the neoplastic process by blocking differentiation of erythroblasts (Shuetze et al, 1993; Moreau-Gachelin et al, 1996). We previously reported that overexpression of the *PU.1* gene in MEL cells blocks cell differentiation, inhibits cell growth and induces apoptosis in the presence of dimethylsulphoxide (DMSO) (Yamada et al, 1997). These results suggest that the gene exerts versatile effects on transformed phenotypes.

In this report, we attempted to investigate the possible effect of PU.1 on the transformed phenotypes other than in haematopoietic cells. We ectopically expressed the *PU.1* gene in a human fibrosarcoma cell line, HT1080, harbouring the activated N-*ras* gene and observed alterations of the biological phenotypes.

*Received 18 August 1997*
*Revised 3 March 1998*
*Accepted 11 March 1998*

*Correspondence to:* T Oikawa

### MATERIALS AND METHODS

### Plasmid construction

A 1.2-kb mouse *PU.1* full-length cDNA (Klemsz et al, 1990), kindly donated by Dr D. Kabat, was cloned into an eukaryotic expression vector, pLRNL (Hung et al, 1988), downstream of the MuLV promoter.

### Cell culture and transfection

Two subclones, NM-1 and cl-2, were isolated from a human fibrosarcoma cell line, HT1080, purchased from the American Type Culture Collection. Cells were maintained in Dulbecco's modified Eagle minimum essential medium (DMEM) supplemented with 10% fetal bovine serum (FBS). The cells were transfected with the *PU.1* expression vector using Lipofectin reagent (Gibco/BRL) and transfectants were selected in the medium containing G418 (1 mg $ml^{-1}$).

### Western blot analysis

Proteins were electrophoresed in a 12% sodium dodecyl sulphate (SDS)-polyacrylamide gel and transferred electrophoretically on to a nylon membrane (Immobilon; Millipore). The blot was probed with anti-PU.1 polyclonal antibody (Santa Cruz). Chemiluminescent signals of the immunoreactive proteins were visualized on an X-ray film using the ECL kit (Amersham).

**Figure 1** Western blot analysis of PU.1 protein in HT1080 NM-1 cells transfected with an expression vector of mouse PU.1 full-length cDNA. PU.1 migrates at 34 kDa (arrow)

**Table 1** Growth properties of HT1080 NM-1 cells transfected with PU.1 expression vector

| Cells | PU.1 protein levels | Doubling time (h) | Colony formation (%)[a] |
|---|---|---|---|
| Controls | | | |
| HT1080 NM-1 | – | 15.0 | 47.7 ± 6.7 |
| Vector | – | 16.0 | 35.5 ± 5.7 |
| PU.1 transfectants | | | |
| NMPU.1-7 | ++ | 15.0 | 17.1 ± 1.4 |
| NMPU.1-8-1 | + | 15.0 | 22.5 ± 4.5 |
| NMPU.1-11-L | +++ | 15.0 | 17.7 ± 3.3 |
| NMPU.1-26 | ± | 15.0 | 20.7 ± 6.3 |

[a]$2.5 \times 10^3$ cells per plate were seeded. Colony formation was scored into duplicated dishes 3 weeks after the seeding.

**Figure 2** Morphology of colonies at the early stage of *PU.1* transfectants in soft agar. Parental HT1080 MN-1 cells (**A** and **C**) and *PU.1*-transfectant NMPU.1-7 cells (**B** and **D**) were cultured in soft agar either in the absence (**A** and **B**) or presence (**C** and **D**) of $10^{-7}$ M DEX as described in Materials and methods

### Cell growth analysis

A total of $1 \times 10^4$ cells were cultured in 5 ml of culture medium in 6-cm plastic dishes. Cell numbers were counted every 24 h and doubling times were calculated by means of cell numbers from triplicated samples in the exponentially growing stage of cells.

### Soft agar colony-formation assay

A total of $2.5 \times 10^3$ cells were plated in 0.3% Noble agar (Difco) in DMEM with 10% FBS in the presence or absence of $10^{-7}$ M of DEX. After 3 weeks of incubation, cells were stained with *P*-iodonitrotetrazolium violet (1 mg $ml^{-1}$) and colonies containing more than 64 cells were counted under a microscope. Morphology of colonies 4 days after cell seeding in soft agar was observed under a phase-contrast microscope at 200 × magnification.

### DNA probes

An *Eco*RI fragment of human N-*ras* cDNA (Taparowsky et al, 1983) was derived from the Japanese Collection of Research Biosources-Gene (Tokyo, Japan). A *Pst*I fragment of human urokinase cDNA (Veder et al, 1984) was derived from the American Type Culture Collection (ATCC; Rockville, MD, USA).

### Northern blot analysis

Total RNA (10 μg) extracted from cells was denatured and electrophoresed in 1.2% agarose gels containing 0.66 M formaldehyde. Hybridization was performed as described (Kondoh et al, 1992) using cDNA probes labelled with [$^{32}$P] α-dCTP by the method of Feinberg and Vogelstein (1983). Specific hybridization was visualized by film autoradiography and band intensity was measured by a densitometer (GS-700, Bio-Rad).

### Nuclear run-on assay

A nuclear run-on assay was performed as described (Kondoh et al, 1991). Filters were blotted with 5 μg of *Pst*I fragment of human urokinase cDNA and 5 μg of *Bam*HI/*Eco*RI fragment of human cardiac actin DNA (Gunning et al, 1984). Specific hybridization was visualized and band intensity was measured by the BAS-2000 image analysing system (Fuji Film, Tokyo, Japan).

### RESULTS

### Expression of PU.1 protein in *PU.1*-transfected HT1080 cells

To examine cell biological function of PU.1, we transfected a pLRNL expression vector containing full-length mouse *PU.1* cDNA into a clone (NM-1) of PU.1-negative human fibrosarcoma HT1080 cells. Four independent PU.1-expressing HT1080 NM-1 clones were obtained and designated NMPU.1-7, NMPU.1-8-1, NMPU.1-11-L and NMPU.1-26 respectively. Western blot analysis using polyclonal antibody against PU.1 revealed that NMPU.1-7 and NMPU.1-11-L cells expressed higher and the highest levels of the protein, whereas NMPU.1-8-1 and NMPU.1-26 cells expressed moderate and the lowest levels of the protein respectively. No PU.1 protein was observed in parental HT1080 NM-1 cells (Figure 1).

### Cell biological features of *PU.1*-transfected HT1080 cells

We first examined the effect of ectopic expression of PU.1 on growth properties of HT1080 NM-1 cells. As shown in Table 1, we found no substantial effect of PU.1 on the doubling time for proliferation of NM-1 cells grown in the adherent culture. However,

© Cancer Research Campaign 1998

**Figure 3** Expression of the *PU.1*, N-*ras* and *uPA* genes in *PU.1*-transfected HT1080 NM-1 (**A**) and cl-2 (**B**) clones and mock-transfected clones (vector). Total RNAs were extracted from the cells grown either in the absence (–) or presence (+) of $10^{-7}$ M DEX for 24 h. The ribosomal RNAs in agarose gels were stained with ethidium bromide for an internal control of sample loading when we examined growth of the cells in soft agar, we found a significant reduction in colony formation in *PU.1*-transfected cells compared with the control cells of parental and mock-transfected NM-1 cells. The degree of reduction of colony-forming ability in the transfectants seemed to be inversely correlated with the degree of the levels of PU.1 protein in these cells (Table 1). Morphological examination of soft-agar colonies at the early stage under a microscope demonstrated that all the *PU.1* transfectants exhibited colonies with cells sparsely distributed. By contrast, parental cells and mock transfectants exhibited colonies with very limited cell spreading in soft agars (Figure 2), suggesting that expression of PU.1 may increase migration of cells in soft agar. Representative morphological appearances of the parental cells and a *PU.1* transfectant (NMPU.1-7) grown in soft agar are shown in Figure 2A and B respectively. Other *PU.1* transfectants also exhibited similar growing patterns to NMPU.1-7 (data not shown). In spite of the difference in growth patterns in soft agar, no particular morphological alterations were observed between *PU.1* transfectants and the control cells grown in plastic culture plates (data not shown).

### Expression of the *uPA* and N-*ras* genes in *PU.1*-transfected HT1080 cells

It has been reported that the activated N-*ras* gene is crucial for keeping the transformed phenotypes of HT1080 cells (Hall et al, 1983; Paterson et al, 1987) and that the elevated levels of expression of urokinase-type plasminogen activator (uPA) are associated with cell migration and malignant transformation in several types of tumour cells (Kirchheimer and Remold, 1989; Chambers et al, 1995). Therefore, we examined whether PU.1 could affect expression of these genes in HT1080 NM-1 cells. As shown in Figure 3A, NMPU.1-7 and NMPU.1-11-L cells expressed higher levels of *PU.1* mRNA transcribed from the viral long terminal repeat (LTR) in the expression vector (a 2-kb transcript and a 4-kb through-reading transcript), NMPU.1-8-1 cells moderate level, and NMPU.1-26 lower level. No expression of the *PU.1* gene was observed in parental and mock-transfected NM-1 cells. The levels of *PU.1* mRNA were almost consistent with the protein levels detected by Western blot analysis shown in Figure 1. Expression of the N-*ras* oncogene was not markedly affected by ectopic expression of PU.1 in NM-1 cells. However, the steady-state levels of uPA mRNA were significantly enhanced in all of the *PU.1* transfectants compared with parental NM-1 cells and a mock transfectant (compare lanes 1 and 3 with lanes 5, 7, 9 and 11 in Figure 3A). This was also true in another series of transfectants (LRPU.1-6, LRPU.1-9, LRPU.1-14 and LRPU.1-15) established by transfecting the *PU.1* expression vector into another HT1080 subclone, cl-2 (compare lanes 1 and 3 with lanes 5, 7, 9 and 11 in Figure 3B).

### Effect of glucocorticoid on uPA expression and cell migration in HT1080 cells

It is certainly evident that synthetic glucocorticoids such as dexamethasone (DEX) act as suppressors of uPA at the transcriptional

 © Cancer Research Campaign 1998

**Figure 4** Nuclear run-on analysis of the *uPA* (U) and actin (A) genes in mock-transfected (vector) and *PU.1*-transfected LRPU.1-15 cells untreated (–) or treated (+) with $10^{-7}$ of DEX for 24 h. The signal specific for the uPA gene was compared as a ratio of the intensity with that of the actin gene used as an internal control level in HT1080 cells (Medcalf et al, 1986). Furthermore, transcriptional interference between PU.1 and steroid/vitamin receptor family members has been reported in vitro using the chloramphenicol acetyltransferase (CAT) assay (Gauthier et al, 1993). Therefore, we examined whether expression of PU.1 interferes with the effect of DEX on uPA expression in vivo in HT1080 cells. As shown in Figure 3A and B, treatment of HT1080 cells and mock transfectants with $10^{-7}$ M of DEX greatly reduced uPA mRNA levels in these cells. Although a decrease in the level of uPA transcripts was also observed in *PU.1*-transfected cells after treatment with DEX, the levels were still higher than those in parental and mock-transfected control cells without DEX treatment. Densitometric analysis revealed that the levels were reduced to 80%, 62%, 73% and 40% in NMPU.1-7, NMPU.1-8-1, NMPU.1-11-L and NMPU.1-26 cells, whereas they were reduced to 40% and 50% in parental and mock-transfected NM-1 cells respectively (Figure 3A). In the same context, DEX reduced the levels of uPA mRNA to 60%, 33%, 55% and 87% in LRPU.1-6, LRPU.1-9, LRPU.1-14 and LRPU.1-15 cells, but to less than 10% in control cl-2 cells, respectively (Figure 3B). In contrast, DEX did not affect N-*ras* gene expression in these *PU.1*-expressing cells as well as the control cells. In HT1080 cells, DEX seemed to increase slightly rather than decrease expression of the transfected *PU.1* gene. These results indicate that DEX specifically reduced uPA expression in HT1080 cells but the degree of reduction was alleviated by expression of the *PU.1* gene.

The effect of DEX on growth properties of *PU.1* transfectants was then examined. Neither cell growth in adherent culture nor colony formation in soft agar of HT1080 cells was inhibited even in the presence of DEX (data not shown). Microscopic examination of the colonies in soft agar showed that parental and mock-transfected HT1080 NM-1 cells formed tight and more compact confines without cell spreading in soft agar in the presence of DEX compared with the colonies in the absence of DEX (compare A with C in Figure 2). On the other hand, *PU.1* transfectants failed to form compact colonies, although cell spreading was much reduced in the presence of DEX (compare C with D, and B with D in Figure 2). These results suggest that DEX reduces cell motility and/or enhances cell–cell adhesion and that ectopic expression of PU.1 alleviates the effect of DEX in HT1080 cells.

### Transcriptional and post-transcriptional control of the uPA gene by ectopic expression of PU.1 and DEX treatment in HT1080 cells

In order to examine whether the effects of PU.1 and DEX on uPA expression occurred at the transcriptional level, a nuclear run-on assay was performed using nuclei isolated from *PU.1*-transfected and mock-transfected cells in the presence or absence of DEX. LRPU.1-15 was chosen as a representative *PU.1* transfectant because of the highest expression of uPA mRNA among the isolated clones. A mock-transfected clone was used as a control. As shown in Figure 4, the levels of uPA transcription were very similar in both cells, although the accumulation of uPA mRNA was significantly higher in LRPU.1-15, compared with mock-transfected cells. The levels of the actin transcription, examined as a control gene, were almost the same between control and LRPU.1-15 cells. Northern blot analysis demonstrated that levels of actin mRNA were also unchanged between the two (data not shown). On the other hand, the relative level of uPA transcription in mock-transfected cells, as judged in comparison with the level of actin transcription, was significantly reduced to 25% by DEX treatment, whereas in LRPU.1-15 cells it was merely reduced to 88%. The actin transcription was not reduced by DEX in both cells. These results suggest that the transcription rate of the uPA gene was unaffected by PU.1 in the absence of DEX and that increase in steady-state level of uPA mRNA by PU.1 is mainly accounted for by post-transcriptional control. Meanwhile, transcription of the uPA gene was markedly reduced by DEX treatment. The negative effect of DEX on the uPA transcription was, however, significantly alleviated by expression of PU.1 in HT1080 cells.

### DISCUSSION

We found that exogenous expression of PU.1 in HT1080 human fibrosarcoma cells increased cell migration in soft agar. In association with these cell biological changes, expression of the uPA mRNA, which encodes a serine protease correlated with cell migration and invasion, was enhanced in *PU.1*-transfected HT1080 cells compared with mock transfectants. The *uPA* gene has a transcriptional enhancer region, which consists of binding sites for Fos/Jun family transcription factors AP-1 and an Ets family transcription factor PEA3, and this region has been reported to be critical for activation of the gene in HT1080 cells (Nerlov et al, 1991). In murine macrophages, Ets-2 activates the uPA enhancer by binding to the PEA3 site, whereas other Ets family transcription factors such as PU.1 and PEA3 do not activate the enhancer (Stacey et al, 1995). To examine whether purified PU.1 protein or that in a crude nuclear extract from LRPU.1-15 cells can bind to the PEA3 site, we performed an electrophoretic mobility shift assay. However, PU.1 did not bind to this site (data not shown), suggesting that PU.1 cannot directly affect the enhancer region of the uPA gene through the PEA3 site within this region. Furthermore, run-on analysis demonstrated that PU.1 did

© Cancer Research Campaign 1998 

not affect uPA gene expression at the transcriptional level in HT1080 cells. Therefore, the accumulation of the uPA mRNA in *PU.1*-transfected cells may be due to the post-transcriptional mechanism(s), perhaps by increasing the uPA mRNA stability in the cells. There are several reports demonstrating post-transcriptional regulation of uPA gene; in rat fibroblasts *uPA* gene expression is post-transcriptionally suppressed compared with highly metastatic carcinoma cells (Henderson et al, 1991, 1992); uPA mRNA stability is specifically increased by a synergistic effect of $Ca^{2+}$ and cAMP in renal epithelial cells (Altus et al, 1987, 1991; Zeigler et al, 1990); and cycloheximide, an inhibitor of protein synthesis, increases the uPA mRNA half-life from 70 min to >20 h (Altus et al, 1991). Therefore, there may be a protein(s) of short-half life, potentially attaching to the A+U-rich elements in the 3′-untranslated region of the uPA mRNA and responsible for specific degradation of the mRNA (Altus et al, 1991; Henderson and Kefford 1991). PU.1 may affect the level and/or function of such proteins.

There are several reports showing that glucocorticoid can lower uPA activity in a number of cell types (Littlefield et al, 1985; Medcalf et al, 1986). Consistent with previous reports by others (Medcalf et al, 1986), our results suggest that the suppression of the *uPA* gene expression by DEX occurs at the transcriptional level in HT1080 cells. Furthermore, we demonstrate that DEX antagonized the effect of PU.1 on morphology of colonies grown in soft agar: PU.1 stimulated cell migration, whereas DEX inhibited it. In parallel with this, PU.1 alleviated the negative effect of DEX on uPA expression at the transcriptional level in HT1080 cells. It has been reported that the glucocorticoid receptor (GR) interferes with the function of PU.1 and vice versa in human breast cancer cells (Gauthier et al, 1993). Similar mutual functional interference between Fli-1, another ETS family protein, and steroid hormone receptors was also reported recently (Darby et al, 1997). Therefore, our observation may be accounted for by such a functional interference, by which PU.1 blocks the transcriptional suppressing activity of GR in HT1080 cells.

We also found that PU.1 brought about negative effects upon the anchorage-independent growth of HT1080 cells: the *PU.1* transfectants exhibited reduced colony formation in soft agar. Similar observations have been reported in some tumours: constitutive expression of Ets-1 reverts the tumorigenicity in human colon cancer cells (Suzuki et al, 1996) and stable expression of a *trans*-dominant mutant of PU.1 or Ets-2 also reverts Ras-transformed NIH/3T3 cells (Langer et al, 1992; Wasylyk et al, 1994). It is known that HT1080 cells harbour the activated N-*ras* oncogene, which is essential for maintaining the transformed phenotypes of the cells, and inhibition of N-*ras* gene expression rendered the cells less malignant (Hall et al, 1983; Paterson et al, 1987). However, *PU.1*-transfected HT1080 cells expressed similar levels of N-*ras* mRNA to the parental cells and mock transfectants (Figure 3A and B). As a member of the Ets family proteins is one of the nuclear targets of the *ras* signalling pathway to activate ras-responsive elements (Wasylyk et al, 1990; Galang et al, 1994), the ectopic expression of PU.1 may perturb endogenous Ets family proteins, which mediate the *ras* signals crucial for maintenance of the transformed phenotypes of HT1080 cells.

In conclusion, our results could provide an insight into a novel function of Ets-related transcription factor PU.1 on several transformed phenotypes provoked, in part, by the activated N-*ras* oncogene.

### ABBREVIATIONS

DEX, dexamethasone; DMEM, Dulbeco's modified Eagle minimum essential medium; DMSO, dimethylsulphoxide; GR, glucocorticoid receptor; MuLV, murine leukaemia virus; LTR, long terminal repeat; SDS, sodium dodecyl sulphate; uPA, urokinase-type plasminogen activator.

### ACKNOWLEDGEMENTS

We thank Dr J Akiyama, OB-GYN Akiyama Memorial Hospital, Hakodate, Japan, for his continuous encouragement and financial support. This work was partially supported by a Grant-in Aid for Scientific Research on Priority Areas to TO from the Ministry of Education, Science and Culture of Japan.

### REFERENCES

Altus MS and Nagamine Y (1991) Protein synthesis inhibition stabilizes urokinase-type plasminogen activator mRNA. *J Biol Chem* **266**: 21190–21196

Altus MS, Pearson D, Horiuchi A and Nagamine Y (1987) Inhibition of protein synthesis in LLC-PK1 cells increased calcitonin-induced plasminogen-activator gene transcription and mRNA stability. *Biochem J* **242**: 387–392

Chambers SK, Wang Y, Gerts RE and Kacinski BM (1995) Macrophage colony-stimulating factor mediates invasion of ovarian cancer cells through urokinase. *Cancer Res* **55**: 1578–1585

Darby TG, Meißner JD, Rühlmann A, Mueller WH and Scheib RJ (1997) Functional interference between retinoic acid or steroid hormone receptors and oncoprotein Fli-1. *Oncogene* **15**: 3067–3082

Feinberg AP and Vogelstein BA (1983) A technique for radiolabeling DNA restriction endonuclease fragments to high specific activity. *Anal Biochem* **132**: 6–13

Galang CK, Der CJ and Hauser CA (1994) Oncogenic Ras can induce transcriptional activation through a variety of promoter elements, including tandem c-Ets-2 binding sites. *Oncogene* **9**: 2913–2921

Gauthier J-M, Bourachot B, Daucaus V, Yaniv M and Moreau-Gachelin F (1993) Functional interaction between Spi-1/PU.1 oncoprotein and steroid hormone or vitamin receptors. *EMBO J* **12**: 5089–5096

Gunning P, Ponte P, Kedes Lhickei PJ and Scoultchi AI (1984) A sarcomeric actin associates with a nonmuscle cytoskeleton. *Cell* **36**: 709–715

Hall A, Marshall CJ, Spurr NK and Weiss RA (1983) Identification of transforming gene in two human sarcoma cells as a new member of ras gene family located on chromosome 1. *Nature* **303**: 396–400

Henderson BR and Kefford RF (1991) Conservation and potential role of RNA-instability motifs in urokinase gene 3′-untranslated sequences. *J Natl Cancer Inst* **83**: 1103–1104

Henderson BR, Tansey WP, Phillip SM, Ramshaw IA and Kefford RF (1992) Transcriptional and posttranscriptional activation of urokinase plasminogen activator gene expression in metastatic tumor cells. *Cancer Res* **52**: 2489–2496

Hung H-JS, Yee J, Shew J, Chen P, Bookstein R, Friedmann T, Lee EY-HP and Lee W (1988) Suppression of the neoplastic phenotypes by replacement of the RB gene in human cancer cells. *Science* **242**: 1563–1566

Kirchheimer JC and Remold HG (1989) Endogenous receptor-bound urokinase mediates tissue invasion of human monocytes. *J Immunol* **143**: 2634–2639

Klemsz MJ, Mckercher SR, Celada A, Van Beveran C and Maki RA (1990) The macrophage and B-cell-specific transcription factor PU.1 is restricted to the *ets* oncogene. *Cell* **61**: 113–124

Kondoh N, Oikawa T, Satoh C and Kuzumaki N (1991) Effects of sodium butyrate on the rearranged *c-myc* expression in mouse plasmacytoma cells. *Exp Cell Res* **196**: 146–149

Kondoh N, Schweinfest CW, Henderson KW and Papas TS (1992) Differential expression of S29 ribosomal protein, laminin-binding protein and human lymphocyte antigen class I messenger RNAs associated with colon carcinoma progression and differentiation. *Cancer Res* **52**: 791–796

Langer SJ, Bortner DM, Roussel MF, Sherr CJ and Ostrowski MC (1992) Mitogenic signaling by colony-stimulating factor 1 and ras is suppressed by the *ets*-2 DNA-binding domain and restored by *myc* over-expression. *Mol Cell Biol* **12**: 5355–5362

© Cancer Research Campaign 1998

Littlefield BA, Johnston LJ, Manzer DS and Roche PC (1985) Glucocorticoid inhibition of urokinase-like plasminogen activators in cultured human lymphoblasts. *Endocrinology* **117**: 1100–1109

Medcalf RL, Richard RI, Crawford RJ and Hamilton JA (1986) Suppression of urokinase-type plasminogen activator mRNA levels in human fibrosarcoma cells and synovial fibroblasts by anti-inflammatory glucocorticoids. *EMBO J* **5**: 2217–2222

Moreau-Gachelin F (1994) Spi-1/PU.1: an oncogene of Ets family. *Biochim Biophys Acta* **1198**: 149–163

Moreau-Gachelin F, Tavitian A and Tambourin P (1988). Spi-1 is a putative oncogene in virally induced murine erythroleukemias. *Nature* **331**: 277–280

Moreau-Gachelin F, Wendling F, Molina T, Denis N, Titeux M, Grimber G, Briand P, Vainchenker W and Tavitian A (1996) Spi-1/PU.1 transgenic mice develop multistep erythroleukemias. *Mol Cell Biol* **16**: 2453–2463

Nerlov C, Rorth P, Blasi F and Johnsen M (1991) Essential AP-1 and PEA3 binding elements in the human urokinase enhancer display cell type-specific activity. *Oncogene* **6**: 1583–1592

Paterson H, Reeves B, Brown R, Hall A, Furth M, Bos J, Jones P and Marshall C (1987) Activated N-*ras* controls the transformed phenotype of HT1080 human fibrosarcoma cells. *Cell* **51**: 803–812

Paul R, Schuetze S, Kozak SL, Kozak CA and Kabat D (1991) The *Sfpi-1* proviral integration site of Friend erythroleukemia encodes the *ets*-related transcription factor PU.1. *J Virol* **65**: 464–467

Schuetze S, Stenberg P and Kabat D (1993) The Ets-related transcription factor PU.1 immortalizes erythroblasts. *Mol Cell Biol* **13**: 5670–5678

Simon MC, Olson M, Scott E, Hack A, Su G and Singh H (1996) Terminal myeloid gene expression and differentiation requires the transcription factor PU.1. *Curr Top Micro Immunol* **211**: 113–119

Stacey KJ, Fowles LF, Colman MS, Ostrowski MC and Hume DA (1995) Regulation of urokinase-type plasminogen activator gene transcription by macrophage colony-stimulating factor. *Mol Cell Biol* **15**: 3430–3441

Suzuki H, Romano-Spica V, Papas TS and Bhat NK (1996) ETS1 suppresses tumorigenicity of human colon cancer cells. *Proc Natl Acad Sci USA* **92**: 4442–4446

Taparowsky E, Shimizu K, Goldfarb M and Wigler M (1983) Structure and activation of the human N-*ras* gene. *Cell* **34**: 581–586

Veder P, Stoppelli MP, Galeffi P, Di Nocera P and Blasi F (1984) Identification and primary sequence of an unspliced human urokinase poly $(A)^+$ RNA. *Proc Natl Acad Sci USA* **81**: 4727–4731

Wasylyk B, Wasylyk C, Flores P, Begue A, Leprine D and Stehelin D (1990) The *ets* proto-oncogenes encode transcription factors that cooperate with c-Fos and c-Jun for transcriptional activation. *Nature* **346**: 191–193

Wasylyk C, Maria S-M, Sobieszczuk P and Wasylyk B (1994) Reversion of Ras transformed cells by Ets transdominant mutants. *Oncogene* **9**: 3665–3673

Yamada T, Kondoh N, Matsumoto M, Yoshida M, Maekawa A and Oikawa T (1997) Overexpression of PU.1 induces growth and differentiation inhibition and apoptotic cell death in murine erythroleukemia cells. *Blood* **89**: 1383–1393

Ziegler A, Hagmann J, Kiefer B and Nagamine Y (1990) $Ca^{2+}$ potentiates cAMP-dependent expression of urokinase-type plasminogen activator gene through a calmodulin- and protein kinase C-dependent mechanism. *J Biol Chem* **265**: 21194–21201

© Cancer Research Campaign 1998